# Differential Function of Themis CABIT Domains during T Cell Development

**DOI:** 10.1371/journal.pone.0089115

**Published:** 2014-02-21

**Authors:** Toshiyuki Okada, Takeshi Nitta, Kentaro Kaji, Akiko Takashima, Hiroyo Oda, Norimasa Tamehiro, Motohito Goto, Tadashi Okamura, Michael S. Patrick, Harumi Suzuki

**Affiliations:** 1 Department of Immunology and Pathology, Research Institute, National Center for Global Health and Medicine, Ichikawa-shi, Chiba, Japan; 2 Department of Laboratory Animal Medicine, Research Institute, National Center for Global Health and Medicine, Shinjuku, Tokyo, Japan; 3 Department of Infectious Diseases, Research Institute, National Center for Global Health and Medicine, Shinjuku, Tokyo, Japan; University of Iowa, United States of America

## Abstract

Themis (also named Gasp) is a newly identified Grb2-binding protein that is essential for thymocyte positive selection. Despite the possible involvement of Themis in TCR-mediated signal transduction, its function remains unresolved and controversial. Themis contains two functionally uncharacterized regions called CABIT (cysteine-containing, all-β in Themis) domains, a nuclear localization signal (NLS), and a proline-rich sequence (PRS). To elucidate the role of these motifs in Themis’s function in vivo, we established a series of mutant Themis transgenic mice on a Themis^−/−^ background. Deletion of the highly conserved Core motif of CABIT1 or CABIT2 (Core1 or Core2, respectively), the NLS, or the PRS abolished Grb2-association, as well as TCR-dependent tyrosine-phosphorylation and the ability to induce positive selection in the thymus. The NLS and Core1 motifs were required for the nuclear localization of Themis, whereas Core2 and PRS were not. Furthermore, expression of ΔCore1- but not ΔCore2-Themis conferred dominant negative-type inhibition on T cell development. Collectively, our current results indicate that PRS, NLS, CABIT1, and CABIT2 are all required for positive selection, and that each of the CABIT domains exerts distinct functions during positive selection.

## Introduction

T cells develop through positive and negative selection in the thymus to become either class II MHC-restricted helper CD4^+^CD8^−^ [CD4-single positive (CD4SP)] or class I MHC-restricted cytotoxic CD4^−^CD8^+^ (CD8SP) cells [1]. However, the molecular mechanisms of TCR-mediated selection in developing T cells are not yet fully understood.

Themis (thymocyte-expressed molecule involved in selection) was identified as a novel mandatory factor for positive selection by five independent groups in 2009 [2-3-4-5-6]. We identified Themis (initially named Gasp) from a set of uncharacterized genes whose expression was restricted to the thymus [2]. Themis knockout mice exhibited significantly reduced numbers of CD4SP and CD8SP T cells both in the thymus and periphery [2-3-4-5-6]. Inhibitory effects of Themis deficiency on negative selection and T cell activation were controversial among the reports [2-3-4-5], nevertheless, they were much slighter than that for positive selection. Thus, Themis is a unique molecule that is essential for positive selection, but not for negative selection. Expression of Themis was significantly lower in regulatory T cells (Tregs) compared to conventional T cells [7], however, study of a natural mutant rat revealed the importance of Themis on the function of Tregs [8]. Since Themis-deficient mice did not exhibit inflammatory bowel disease or autoimmune diseases observed in Treg-deficient mice, functional requirements for Themis in Tregs could be different between rat and mouse.

Themis is constitutively associated with Grb2 and is tyrosine-phosphorylated by Lck and ZAP-70 upon TCR stimulation [5-9-10]. Some groups reported constitutive association of Themis with Vav1 [9], Itk, and PLC-γ1 [5-10-11]. Furthermore, we and other groups (Fig. S3) demonstrated association of Themis with PLC-γ1, LAT, and SLP76 [5-9-10-11] upon TCR-stimulation, indicating that Themis would be a component of the SLP76-LAT signalosome. From these results, Themis is likely to be involved in TCR-mediated signal transduction. Accordingly, TCR-dependent activation of ERK and NFAT, as well as production of IL-2 was significantly reduced in Themis knockdown Jurkat cells [10]. In the Themis-knockout mice, however, results were different. We and other groups observed unaltered activation of ERK and calcium influx in Themis deficient immature DP thymocyte upon anti-CD3 antibody stimulation [2-3-4], although one group reported impaired activation of these signaling events [5]. Moreover, recent study showed that TCR-dependent activation of ERK, p38 and Vav1 were reduced in Themis deficient CD4SP and CD8SP thymocytes [9]. After all, results were not consistent between different groups possibly because of different experimental systems, and therefore no consensus has been reached about the effect of Themis on TCR-mediated signal transduction.

Themis contains two novel cysteine-based CABIT (cysteine-containing, all beta in Themis) domains [4], a bipartite type nuclear localization sequence (NLS), and a proline-rich sequence (PRS). The CABIT domain is a newly designated domain structure conserved among metazoans, and it could adopt an all-beta-strand structure with at least 12 strands, which suggests either an extended beta-sandwich-like fold or a dyad of six-stranded beta-barrel units [4]. In mammals, the CABIT domains are conserved among three Themis family proteins (Themis/Themis1, ICB1/Themis2 and 9130404H23Rik/Themis3) harboring two tandemly-repeated CABIT domains (CABIT1 and CABIT2) and two Fam59 proteins (Fam59a and b) containing one CABIT domain [4]. Although a number of proteins containing CABIT domains have been identified, their function is totally unknown. Therefore, elucidation of the function of CABIT domains has long been awaited.

In order to reveal the function of Themis in positive selection, we investigated the function of each structural domain and motif in Themis. In the present study, we generated a series of transgenic (Tg) mice expressing mutant Themis proteins lacking each domain on a Themis^−/−^ background. Deleted motifs were the PRS and the highly conserved cysteine-containing Core motif of the CABIT1 or CABIT2 domain (Core1 and Core2, respectively). We found that the PRS, Core1, and Core2 motifs were all required for Grb2-binding and TCR-dependent tyrosine-phosphorylation, as well as for positive selection in the thymus. Interestingly, the Core1 and NLS motifs were required for nuclear localization of Themis, whereas Core2 and PRS were not. Furthermore, Core1- but not Core2-deleted mutant exhibited a dominant negative inhibitory effect on T cell development even in the presence of wild-type Themis. These results indicate that each structural motif in Themis exerts an essential but distinct role in T cell development, and that the two CABIT domains in Themis have distinct functions.

## Materials and Methods

### Mice

Themis^−/−^ mice have been previously described [2]. For generation of Themis transgenic mice, PCR-cloned cDNA fragments encoding WT or ΔPRS,ΔNLS, ΔCore1, ΔCore2, or CAB2-1 mutants of Themis were inserted into the hCD2-VA vector [12]. The transgenes were purified and microinjected into the pronuclei of fertilized eggs from mixed background mice [BDF1 (SLC)×Themis^−/−^ mice on a C57BL/6 background] using standard procedures. The embryos were transferred to the oviducts of pseudopregnant ICR female mice. Previous studies have shown the human CD2 promoter/enhancer directs the expression of transgenes in mice to the T cell lineage [12]. The ΔPRS, ΔCore1, and ΔCore2-Tg mice used in the study were homozygous for transgene (ΔPRS-Tg^+/+^, ΔCore1-Tg^+/+^, ΔCore2-Tg^+/+^) with the exception of ΔCore1-Tg^+/−^ in the Fig. S4. Instead, the ΔNLS-, WT, and CAB2-1-Tg mice were heterozygous for transgene (ΔNLS-Tg^+/−^, WT-Tg^+/−^, CAB2-1-Tg^+/−^). All mice were housed under specific pathogen-free conditions. All animal experiments were approved by the Animal Care and Use Committee of the National Center for Global Health and Medicine (NCGM) Research Institute, and conducted in accordance with institutional procedures.

### TCR Stimulation

Thymocytes were pre-treated with biotin-conjugated anti-CD3ε (145-2C11, Biolegend) and anti-CD4 (RM4-4, eBioscience) antibodies (10 µg/mL each) at 4°C for 30 min. Cells were then washed, resuspended in RPMI-1640 complete medium, and stimulated with streptavidin (BECKMAN COULTER, 10 µg/mL) at 37°C for the indicated times. Ice-cold PBS or paraformaldehyde (final concentration of 2%) was added to stop stimulation.

### Immunoprecipitations and Western Blot Analysis

Cells were lysed with lysis buffer (50 mM Tris-HCl [pH7.5], 150 mM NaCl, 10 mM MgCl_2_, 0.5% Nonidet P-40) containing protease and phosphatase inhibitor cocktail (Thermo Scientific)). Cell lysates were immunoprecipitated with antibodies conjugated to protein G-sepharose beads (GE). Antibodies used for immunoprecipitation were anti-Themis pAb (06-1328, Millipore) and anti-Themis mAb 2E7. 2E7 is a rat monoclonal antibody produced against recombinant full-length Themis from mice [2]. Antibodies used for western blotting are as follows: anti-Grb2 (MS-20-3, MBL), anti-phospho-tyrosine 4G10 (05-321, Millipore), anti-PLCγ-1 (2822, Cell Signaling), anti-LAT (9166, Cell Signaling), anti-CRK (610035, BD), anti-PARP (ab6079, Abcam), anti-SOS1/2 (SC-259, SantaCruz), and anti-HA (3F10, Roche). Horseradish peroxidase-conjugated anti-IgG secondary antibodies against rabbit, rat, or mouse IgG (GE) were used with Lumigo substrate (Cell signaling).

### Flow Cytometry

For cell-surface staining, the following Abs were used: anti-CD25 (PC61), anti-CD62L (MEL-14), anti-CD4 (RM4-5 and GK1.5), anti-CD8α (5H10-1 and 53-6.7), anti-Foxp3 (FJK-16a), anti-TCRβ (H57-597), anti-CD69 (H1.2F3), anti-CD44 (IM7) Abs. All antibodies were purchased from eBioscience or Biolegend. To examine ERK phosphorylation, cells were fixed and permeabilized with 90% methanol and stained with anti-phospho-ERK (197G2, Cell Signaling) Ab. Foxp3 staining was performed using the FOXP3 staining kit (00-5523-00, eBioscience) according to the manufacturer’s protocol. Cells were analyzed using a FACS CantoII (Becton Dickinson), and the data were analyzed using FlowJo software (Tree Star).

### Subcellular Protein Fractionation

Nuclear and cytoplasmic fractions were isolated from total thymocytes or sorted DP thymocytes using the Subcellular Protein Fractionation Kit (Pierce) following the manufacturer’s protocol.

### Immunofluorescence Confocal Microscopy

Thymocytes were fixed with 2% paraformaldehyde and permeabilized with 0.1% saponin, and stained with anti-Themis antibody (06-1328, Millipore) or isotype control antibody, followed by staining with Alexa-fluor 647-conjugated anti-rabbit IgG (A21246, Molecular Probes, 5 µg/mL) and DAPI (0.2 µg/mL). Images were collected with an Olympus FV-1000 multitracking laser scanning confocal microscope (Olympus Japan) with a 100 × oil objective (NA 1.4), giving a resolution of 0.9 µm in the X, Y – plane.

### Statistical Analysis

All data are represented as means ± SEM. Flow cytometric data were analyzed using the Unpaired T test (GraphPad Prism version 5.0). Asterisks in all figures are as follows: *P<0.05; **P<0.01; ***P<0.001. N.S.; not significant.

## Results

### Establishment of Mutant Themis Transgenic Mouse Lines

**Figure 1:**
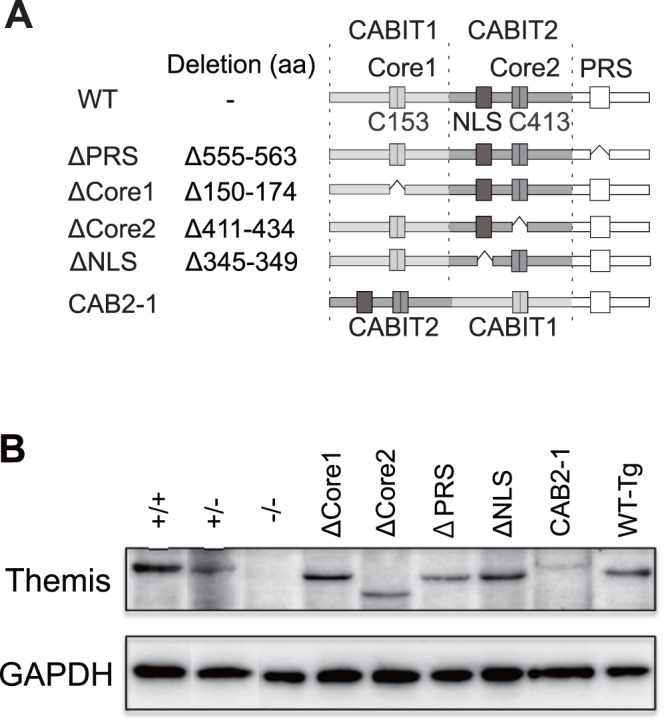
Generation of Themis^−/−^ mice expressing mutant Themis proteins. (A) Schematic representation of the mutant Themis proteins. (B) Analysis of Themis protein expression by immunoblot using sorted CD69^−^ DP thymocytes from Themis^+/+^, Themis^+/−^, Themis^−/−^ and Themis^−/−^ mice expressing ΔPRS, ΔCore1, ΔCore2, ΔNLS, CAB2-1 mutant, or WT Themis. Data are representative of more than three independent experiments.

**Figure 2:**
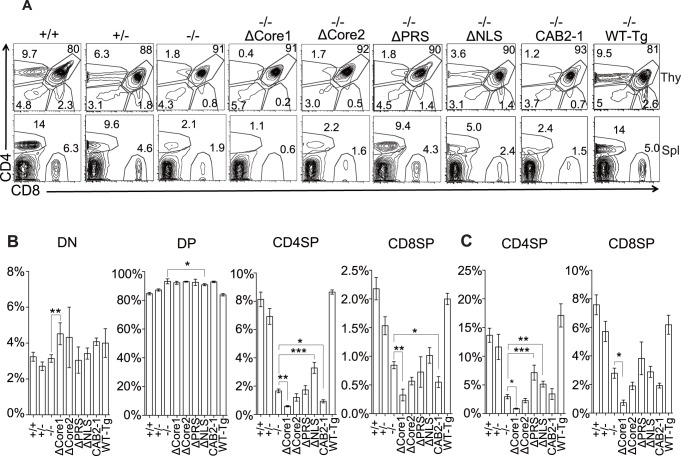
Expression of the Themis mutants failed to restore positive selection in Themis^−/−^ mice. (A) CD4 and CD8 expression profiles of thymocytes or splenocytes from Themis mutant mice. Numbers in plots show the frequency of cells in the indicated area. Proportion (%) of indicated subsets of (B) thymocytes and (C) splenocytes (mean ± SEM). (A-C) Data are representative of more than three independent experiments. Only significant differences against −/− mice are noted in the graphs. *p<0.05, **p<0.01, ***p<0.001.

To determine whether the structural motifs of Themis contribute to its function, we generated a series of transgenic mice expressing mutant Themis proteins lacking each domain (Fig. 1A, Fig. S1). The Themis mutants used in the present study were as follows: ΔPRS (lacking the PRS: aa 555-563), ΔNLS (lacking the NLS: aa 345-349), ΔCore1 (lacking the Core region of CABIT1 domain: aa 150-174), ΔCore2 (lacking the Core region of CABIT2 domain: aa 411-434), and CAB2-1 (swapping entire region of CABIT1 [aa 1-261] and CABIT2 [aa 262-521]). These mutant proteins were expressed under the control of the human CD2 promoter [12]. Each Tg line was then bred with Themis^−/−^ mice to replace endogenous wild-type Themis with each mutant Themis. As shown by Western blotting analysis in Fig. 1B, expression of mutant Themis protein in each Tg line was almost comparable to the endogenous Themis^+/+^ level, although expression of ΔCore2 and CAB2-1 were more comparable to Themis^+/−^ level. As has been reported [9], introduction of a CD2-driven wild-type Themis transgene into Themis^−/−^ mice successfully recovered defective positive selection in the knockout (Fig. 2A), proving that this transgenic approach is useful to evaluate the function of Themis mutant proteins *in vivo*.

### The PRS, NLS, Core1, and Core2 Motifs in Themis are all Required for Positive Selection

First, we analyzed positive selection using these mutant mice. Themis^−/−^ mice showed a marked reduction of CD4SP and CD8SP thymocytes and splenocytes (Fig. 2A) as has been reported [2-3-4-5-6]. Although the transgenic expression of WT Themis completely restored T cell development in Themis^−/−^ mice (Fig. 2A), none of the mutants (ΔPRS, ΔNLS, ΔCore1, ΔCore2, CAB2-1) could restore positive selection. In the thymus, generation of CD4-SP in these mutants was the same or even less than the knockout (Fig. 2A–B, Fig. S2A) with the exception of the NLS mutant which showed some recovery of CD4-SP thymocytes (Fig. 2A–B). In the ΔPRS mutant, peripheral CD4SP cells were significantly recovered despite the defective positive selection in the thymus, suggesting that PRS might not be critical in peripheral expansion and survival of mature T cells. The NLS mutant also showed some recovery of CD4-SP in the periphery, possibly because of the milder defect of positive selection in the thymus. Interestingly, ΔCore1 mutant mice exhibited a severer inhibition of T cell development than the Themis deficient mice. These mice also showed decreased numbers of thymic DP, which was not seen in Themis knockout mice. Also, post-selected (CD69^+^, TCR^hi^) DP thymocytes were significantly fewer in the ΔCore1 mutant mice (Fig. S2C). Indeed, all of the T cell subsets in ΔCore1 Tg mice were fewer than the knockout (Fig. 2B). From these results, loss of the Core1 motif in Themis resulted in a severer phenotype than the loss of entire Themis. Finally, complete loss of positive selection in CAB2-1 mutant indicates that even the order of the two CABIT domains is critical for its function. Collectively, the PRS, NLS, Core1, and Core2 are all required for Themis’s function, and each motif may have different roles.

### PRS, NLS, CABIT1, and CABIT2 are Required for Tyrosine-phosphorylation and Grb2-association

**Figure 3:**
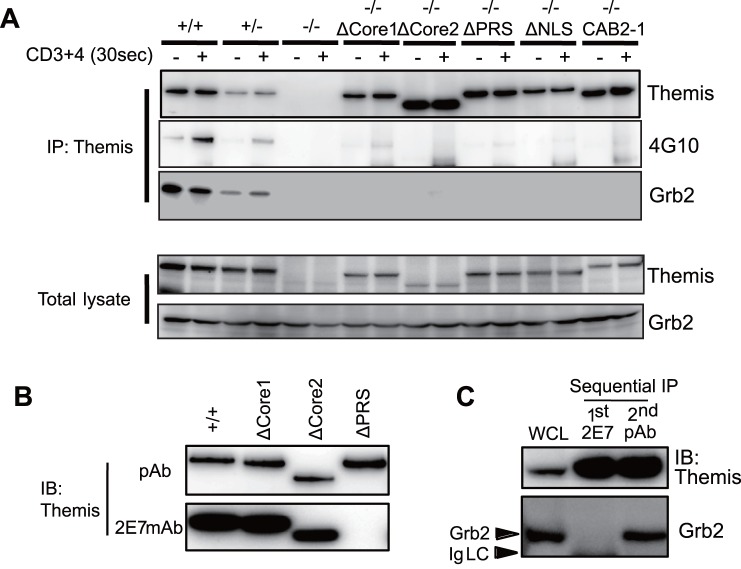
Themis mutants lack tyrosine-phosphorylation and Grb2-association. (A) Analysis of tyrosine-phosphorylation and protein interactions of Themis mutants. Thymocytes from the indicated mutant mice were stimulated with anti-CD3 plus anti-CD4 antibodies. Proteins were immunoprecipitated (IP) with anti-Themis antibody and analyzed by immunoblot (IB) with the indicated antibodies. Data are representative of four independent experiments. (B) Anti-Themis monoclonal antibody (mAb) 2E7 binds to the PRS motif. Immunoprecipitated Themis proteins from Themis^+/+^, Themis^−/−^ ΔCore1, Themis^−/−^ ΔCore2 and Themis^−/−^ ΔPRS thymocytes were immunoblotted with anti-Themis mAb 2E7. Data are representative of three independent experiments. (C) Cell lysates from Themis^+/+^ thymocytes were sequentially immunoprecipitated with 2E7 and anti-Themis polyclonal antibody (pAb). Grb2 was co-precipitated with anti-Themis pAb but not with 2E7. Data are representative of three independent experiments.

We and other groups previously reported that Themis constitutively associates with Grb2 [2], [3], [9]-[11], and is tyrosine-phosphorylated upon TCR stimulation [5], [9]-[11]. We then investigated whether the deletion of each motif affects Grb2-association and tyrosine-phosphorylation. We observed that endogenous wild-type Themis constitutively associated with Grb2 in thymocytes, and was tyrosine-phosphorylated upon stimulation with anti-CD3 plus anti-CD4 antibodies (Fig. 3A) as previously reported [2]-[5], [9]-[11]. In addition, we observed stimulation-dependent association of Themis with PLC-γ1 and SOS (Fig. S3A), suggesting a possible involvement of Themis as a component of the SLP76/LAT-signalosome complex [11]. As shown in Fig. 3A, we could successfully immunoprecipitate ΔPRS, ΔNLS, ΔCore1, ΔCore2, and CAB2-1 Themis mutant proteins, but all of these mutant Themis proteins lost constitutive association with Grb2. Additionally, all five mutants also lost tyrosine-phosphorylation upon TCR stimulation (Fig. 3A). These results indicate that the PRS, NLS, Core1, and Core2 motif, as well as order of the two CABIT domains are all critical for Grb2-association and tyrosine-phosphorylation of Themis.

Because a previous *in vitro* study reported that Themis binds to Grb2 via its PRS motif [11], inability of ΔNLS, ΔCore1, ΔCore2 and CAB2-1 mutants to associate with Grb2 was quite surprising. Therefore, we decided to reinvestigate the biochemical basis of the Themis-Grb2 interaction in thymocytes. We utilized our monoclonal antibody (mAb) against Themis (clone 2E7) [2], which recognizes an epitope around the PRS (aa 555-563) (Fig. 3B). We found that the 2E7 mAb binds only to Grb2-unbound Themis, but not to Grb2-bound Themis by sequential immunoprecipitation experiments (Fig. 3C). These results strongly indicate that 2E7 mAb and Grb2 compete for binding to the PRS, supporting direct association of Grb2 with the PRS of Themis in thymocytes.

### The Core1 but not Core2 Motif is Required for Nuclear Localization

**Figure 4:**
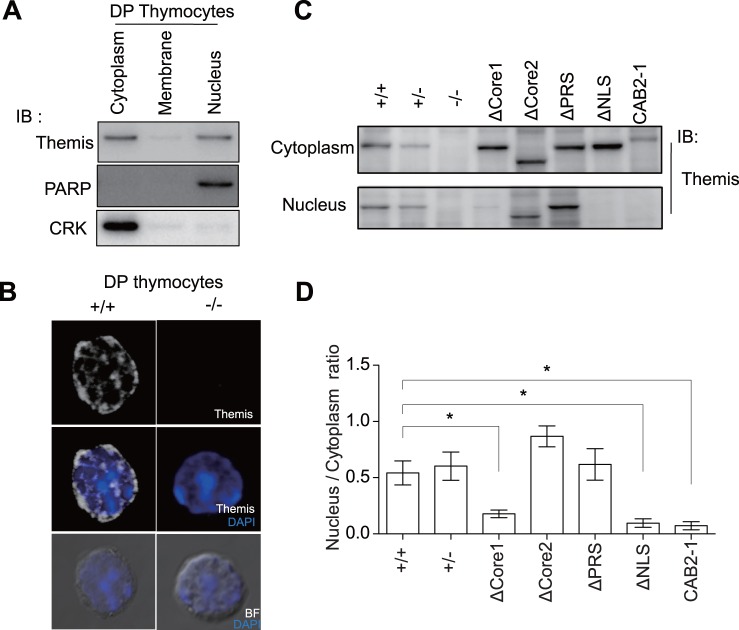
The CABIT1 domain of Themis is important for nuclear localization. (A) DP-thymocytes from Themis^+/+^ mice were fractionated into cytoplasm, membrane and nuclear fractions and immunoblotted with indicated Abs. The CRK and PARP proteins were used as purity controls for the cytoplasmic and nuclear fractions, respectively. Data are representative of more than four independent experiments. (B) Representative micrograph showing localization of Themis (white) in cytoplasm and nucleus stained with DAPI (blue), together with BF (bright field). Data are representative of three independent experiments. (C) Localization of Themis mutants was analyzed by Western blot in cytoplasmic and nuclear extracts. Thymocytes from Themis mutant mice were fractionated into the cytoplasm and nucleus. (D) Bar graph shows that the nuclear/cytoplasmic Themis protein ratio compared with Themis^+/+^ mice. Data are representative of more than three independent experiments. Significant differences are noted in the graphs. *p<0.05.

Although initial experiments using a GFP-fusion protein of Themis exhibited cytosolic localization of Themis protein [2]-[4], Western-blotting results by subcellular fractionation proved that endogenous Themis exists in the nucleus as well as the cytosol [3]-[9]. In fact, we also confirmed the existence of endogenous Themis protein in nuclear fractions by Western blotting (Fig. 4A). Furthermore, existence of Themis in the nucleus was also confirmed by immunostaining analysis by confocal fluorescence microscopy. As shown in Fig. 4B, Themis was visualized in cytosol, as well as in the nucleus as punctate staining. Staining of DNA and Themis appeared mutually exclusive, suggesting that Themis exists unassociated with DNA in the nucleus. We next investigated nuclear localization of each mutant Themis protein in thymocytes by subcellular fractionation. As shown in Fig. 4C-D, ΔPRS and ΔCore2 mutant Themis successfully translocated to the nucleus similar to wild-type Themis, whereas ΔCore1 and ΔNLS mutant existed only in the cytosol (Fig. 4C-D). Although the importance of the NLS motif on nuclear localization of Themis protein has been reported by another group [9], we further showed the Core1 motif to be essential for nuclear translocation of Themis. Furthermore, the order of the two CABIT domains was also critical for the translocation. From these results, we conclude that not only NLS but also the Core motif in the CABIT-1 domain are important for nuclear translocation of Themis.

### Dominant Negative Inhibitory Effect of ΔCore1 Mutant on T Cell Development

**Figure 5:**
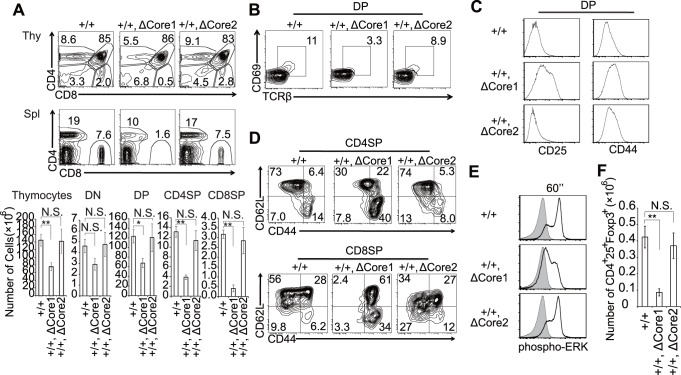
The CABIT1 and CABIT2 in Themis have different functions. (A) CD4 and CD8 profiles of thymocytes and splenocytes of Themis^+/+^, Themis^+/+^ ΔCore1, and Themis^+/+^ ΔCore2 mice. Numbers in plots show the frequency of cells in the indicated area. Bar graphs show absolute number of total thymocytes and thymocyte subsets from Themis^+/+^, Themis^+/+^ ΔCore1, and Themis^+/+^ ΔCore2 mice (mean ± SEM). Data are representative of four independent experiments. (B) Proportion of post-selected CD69^+^ TCR^hi^ cells in CD4^+^CD8^+^ (DP) thymocytes. Data are representative of four independent experiments. (C) Surface expression of CD25 and CD44 on gated DP thymocytes. Data are representative of four independent experiments. (D) CD44 and CD62L profile of splenic CD4SP and CD8SP cells. Data are representative of four independent experiments. (E) Representative histogram overlays of phosphorylation of ERK. DP thymocytes were stimulated with anti-CD3 plus anti-CD4 Abs for 1min, then intracellular staining of phosphorylated-ERK antibody was performed (solid line). The shaded line is without stimulation. Data are representative of three independent experiments. (F) Absolute number of thymic CD4^+^25^+^Foxp3^+^ Treg cells (mean ± SEM). Data are representative of four independent experiments. Significant differences are noted in the graphs. *p<0.05, **p<0.01. N.S. = not significant.

We observed that ΔCore1 mutant Tg mice in Themis^−/−^ background showed severer and distinct defects on T cell development when compared to Themis^−/−^ mice (Fig. 2A). These results indicate that ΔCore1 mutant protein could affect T cell development in a dominant negative fashion. To confirm this possibility, we investigated the phenotype of ΔCore1 Tg mice on a Themis^+/+^ background. Indeed, positive selection of both CD4SP and CD8SP thymocytes was significantly decreased in ΔCore1-Tg Themis^+/+^ mice, whereas ΔCore2-Tg Themis^+/+^ mice did not exhibit any significant change (Fig. 5A). Absolute numbers of DP thymocytes in ΔCore1-Tg Themis^+/+^ were also reduced, which is totally different from the phenotype of Themis deficiency. In addition, numbers of peripheral CD4SP and CD8SP cells were severely reduced in ΔCore1-Tg mice. In ΔCore1-Tg Themis^+/+^ mice we also observed a strong reduction of the earliest post-selected DP (CD69^hi^ TCR^hi^) thymocytes, whereas the reduction was slight in the ΔCore2-Tg Themis^+/+^ mice (Fig. 5B). Expression of CD25 and CD44 on DP thymocytes was somehow increased in ΔCore1-Tg, but not in ΔCore2-Tg Themis^+/+^ mice (Fig. 5C). In the periphery of ΔCore1-Tg Themis^+/+^ mice, memory CD4SP and CD8SP cells (CD44^hi^ CD62L^lo^) were strongly increased with a concomitant decrease of naive populations (CD44^lo^ CD62L^hi^), possibly because of homeostatic expansion in lymphopenic mice (Fig. 5D). Again, this increase of the memory population was observed only in ΔCore1-Tg, but not in ΔCore2-Tg Themis^+/+^ mice. Since ΔCore1-Tg Themis^+/+^ mice showed a strong defect in positive selection, we investigated TCR-dependent activation of ERK [13], which is critical in the process. As shown in Fig. 5E, anti-CD3 plus anti-CD4 mAb stimulated phosphorylation of ERK was strongly inhibited in the presence of ΔCore1 mutant Themis but not of ΔCore2 mutant. Therefore, decreased positive selection in ΔCore1-Tg mice could partly be due to the impaired ERK activation. We also found that the absolute number of CD25^+^Foxp3^+^ natural regulatory T cells (nTreg) in the thymus was significantly reduced in ΔCore1-Tg, but not in ΔCore2-Tg Themis^+/+^ mice (Fig. 5F). Differentiation of NKT and γδ-T cells in Themis^+/+^ ΔCore1 mice was not changed (data not shown). Collectively, expression of Core1-deleted Themis in immature thymocytes caused dominant-negative type inhibition of T cell development, possibly by interfering with signaling related to ERK activation. All of these analyses were carried out using transgene homozygous (ΔCore1-Tg^+/+^ or ΔCore2-Tg^+/+^) mice. Because expression levels of mutant protein in ΔCore2-Tg^+/+^ were significantly less than that of ΔCore1-Tg^+/+^ mice, one can assume that the absence of dominant negative effects in ΔCore2-Tg mice was simply due to the fewer expression of less ΔCore2 mutant protein. Therefore, we compared transgene heterozygous (ΔCore1-Tg^+/−^) mice with homozygous (ΔCore2-Tg^+/+^) mice. As shown in Fig. S4A, protein expression level of mutant Themis protein in ΔCore1-Tg^+/−^ mice and ΔCore2-Tg^+/+^ mice were the same, which were equivalent with that of Themis^+/−^ mice. Although expression of the two mutant proteins were the same level, only ΔCore1-Tg showed dominant negative effects (Fig. S4B-C), proving that the effect is specific to the ΔCore1 mutant protein. Since the dominant negative inhibitory effects were only observed in ΔCore1-Tg but not in ΔCore2-Tg, our current results clearly indicated distinct functions for CABIT1 and CABIT2 domains.

## Discussion

Themis contains an NLS, PRS, and two CABIT domains. The CABIT domain, which was identified by Schwartz’s group, is conserved through protozoa and is predicted to form either an extended beta-sandwich-like fold or a dyad of 6-strand beta-barrel structures [4]. Themis family proteins (Themis, Themis2, and Themis3) contain two CABIT domains and a PRS. A recent report demonstrated that Themis and Themis2 were functionally interchangeable in terms of T cell development [9], indicating the important function of CABIT domains. Therefore, in the present study, we focused on the function of CABIT domains, especially on differences in the two CABIT domains in Themis.

To elucidate the involvement of each domain/motif in Themis on its function in positive selection, we established several mutant Themis Tg lines. Because Themis^+/−^ mice showed an intermediate effect on positive selection between Themis^+/+^ and Themis^−/−^ mice [2], it is obvious that the expression level of Themis protein in thymocytes critically affects the result of positive selection. Therefore, the expression level of mutant protein in these Tg mice is important for evaluating their function. We screened a large number of founder lines of each mutant transgene and managed to establish lines with comparable protein expression (Fig. 1B).

Very recently, the PRS motif was reported to be important for Grb2-association, tyrosine-phosphorylation, and positive selection, using irradiation-mediated chimeric mice reconstituted with retrovirally transduced bone marrow cells [11]. It would be difficult to evaluate the expression level of mutant Themis protein in reconstituted thymocytes, however, our present results using ΔPRS-Tg mice with equivalent protein expression with endogenous Themis (Fig. 1B) strongly supported their observations. Despite strong inhibition of positive selection in the thymus of ΔPRS mice, the number of CD4SP in the periphery was significantly increased compared to Themis^−/−^ mice, suggesting that the PRS motif in Themis is critical in positive selection in the thymus, but may not be important in peripheral survival and maintenance of mature T cells.

We firstly demonstrated that the consensus motif of CABIT2 domain (Core2) was crucial for positive selection. Furthermore, deletion of the Core1 motif resulted in a much severer inhibition of T cell development than seen in Themis^−/−^ mice. Therefore, we next investigated the phenotype of ΔCore1- and ΔCore2-Tgs on a wild-type background. The ΔCore1-Tg mice in Themis^+/+^ background showed decreased numbers of DP thymocytes, strong inhibition of CD8SP thymocyte development, reduced numbers of thymic nTregs, and an increased memory population (CD44^hi^ CD62L^lo^) of peripheral mature T cells. The DP thymocytes from ΔCore1-Tg showed decreased ERK activation upon TCR stimulation. These phenotypes were not observed in Themis^−/−^ mice, suggesting that the ΔCore1 mutant inhibits T cell development in a Themis-independent manner. Instead, the ΔCore1 mutant may perturb the function of unidentified protein(s) that compensate the residual T cell development in Themis^−/−^ mice. Lastly, and most importantly, the dominant negative inhibitory effects were not observed in either the ΔCore2-Tg mice or the wild-type Themis-Tg mice, indicating that the loss of the Core motif of either CABIT1 or CABIT2 domains induces qualitatively different effects. It is possibly explained by differential protein association with ΔCore1 and ΔCore2, or because of the lack of nuclear interaction of ΔCore1 with another protein. Because two independent lines of ΔCore1 Tg mice showed the identical phenotype, the phenotype must not be an artifact caused by the destruction of other genes by the insertion of Tg vector. The dominant negative effects were not due to higher protein expression of the ΔCore1 versus the ΔCore2 mutant, because heterozygous ΔCore1-Tg+/− mice, having equivalent protein expression with homozygous ΔCore2-Tg+/+ mice, also showed the same dominant negative phenotypes (Fig. S4A–C). Collectively, in the present study, we clearly demonstrated differential functions for CABIT1 and CABIT2 domains.

We demonstrated that Themis constitutively associates with Grb2 in thymocytes. Themis2 also associates with Grb2 [9], therefore Grb2-association must be an important feature of Themis family proteins. In the recent peptide inhibition study, the PRS motif of Themis was shown to directly interact with the C-terminal SH3 motif of Grb2 [11]. The result from immunoprecipitation experiments using our own anti-Themis monoclonal antibody (2E7) also supported direct binding of Grb2 to the PRS motif of Themis (Fig. 3B). As has been recently reported [9], we also observed defective association of the ΔPRS mutant Themis with Grb2 in thymocytes (Fig. 3A). More surprisingly, not only ΔPRS, but also all other mutants lost the ability to associate with Grb2 (Fig. 3A). These results suggest that the intra-molecular interaction of these domains is crucial to form the proper three-dimensional structure to associate with Grb2. Moreover, all of the five mutants in the present study lost not only Grb2 binding, but also tyrosine-phosphorylation. It should be noted that these five mutants also lost weak basal phosphorylation of the protein in unstimulated thymocytes (Fig. 3A).

From our sequential immunoprecipitation experiments, a half of Themis binds to Grb2 (Fig. 3C, Fig. S3C), and about one tenth of Grb2 binds to Themis (Fig. S3B). A recent study with T cell-specific Grb2-deficient mice revealed that Grb2 is critical for positive selection [14]. Although Grb2 has been reported to associate with several molecules (e.g. Sos, Shc, FAK) other than Themis [15]-[17], deficiency of Shc or FAK does not inhibit positive selection. Involvement of Sos in positive selection was also recently shown to be nonobligatory because Sos1/Sos2 DKO exhibited normal positive selection [18]. Therefore, Themis and Grb2 may be cooperatively required for positive selection. Further analysis of Themis/Grb2 DKO would clarify whether Themis and Grb2 are functionally complementary during positive selection.

We demonstrated that the PRS, NLS, Core1, and Core2 motifs were all required for Grb2-association, tyrosine-phosphorylation, and positive selection, whereas NLS and CABIT1 are required for nuclear translocation. The CABIT1 domain might be important for the interaction of Themis with nuclear transporter proteins. So far, the causal relationship between positive selection, Grb2-association, and tyrosine-phosphorylation is not yet understood, however, our results demonstrated that all of these are tightly correlated to one another. On the contrary, the current study demonstrated that positive selection and nuclear translocation of Themis are not directly correlated. As a matter of fact, it should be noted that deletion of the NLS motif exhibited the least effect on positive selection compared to the deletion of other motifs, indicating that nuclear translocation of Themis may be less important for positive selection.

Finally, this is the first report that describes the significance of the CABIT domain in cellular events. Furthermore, two CABIT domains exist in Themis are functionally different and not interchangeable. Although the structural basis for the function of CABIT domains remains to be elucidated, our study clearly demonstrated that the two CABIT domains in Themis are pivotal and serve distinct roles in its function for driving T cell development.

## Supporting Information

Figure S1 The amino acid sequence used for generation of mutant Themis transgenes.
(EPS)

Figure S2 Characterization of Themis transgenes lacking the Core1, Core2, PRS, or NLS motif in Themis^−/−^ background mice. Bar graphs represent numbers of (**a**) thymocyte and (**b**) splenocyte subsets (mean ± SEM). (**c**) Proportion of post-selected CD69^+^TCRβ^hi^ DP cells in each mutant. Results are representative of more than three independent experiments. Only significant differences in mutant-Tgs against −/− are noted in the graphs. *p<0.05, **p<0.01, ***p<0.0001.
(EPS)

Figure S3 Themis interacts with the LAT signalosome complex in thymocytes. (**a**) Thymocytes from Themis^+/+^ and Themis^−/−^ mice were stimulated with anti-CD3/anti-CD4/anti-CD8 Abs for indicated times at 37°C. Proteins were immunoprecipitated with anti-Themis pAb and analyzed by immunobloting with the indicated Abs. (**b**) Immunoprecipitates with anti-Themis Ab from Themis^+/+^ thymocytes were immunoblotted with anti-Grb2. Bar graphs show the relative density of the immunoblots of Grb2 from total lysate, immunoprecipitates with anti-Themis (IP), and the supernatant after the immunoprecipitation (Sup). (**c**) Cell lysates from Themis^+/+^ thymocytes were sequentially immunoprecipitated with 2E7 and anti-Themis Ab. Bar graphs show the relative density of the immunoblot Themis bands (mean ± SEM). Results are representation of three independent experiments. The relative densities are expressed as mean ± SEM.
(EPS)

Figure S4 Dominant-negative inhibitory effect observed in Themis^+/+^ ΔCore1-Tg^+/−^ mice. (**a**) Comparison of protein expression by immunoblot between thymocytes from Themis^+/+^ ΔCore1-Tg^+/−^ and Themis^+/+^ ΔCore2-Tg^+/+^. (**b**) CD4 and CD8 expression profiles of thymocytes from Themis^+/+^ and Themis^+/+^ mice expressing heterozygous ΔCore1-Tg^+/−^ and Themis ΔCore2-Tg^+/+^. Numbers in plots show the frequency of cells in the indicated area. Results are representative of three independent experiments. (**c**) Proportion of post-selected CD69^+^TCRβ^hi^ DP cells in each mutant. Results are representative of three independent experiments.
(EPS)
